# Two-Swim Operators in the Modified Bacterial Foraging Algorithm for the Optimal Synthesis of Four-Bar Mechanisms

**DOI:** 10.1155/2016/4525294

**Published:** 2016-02-28

**Authors:** Betania Hernández-Ocaña, Ma. Del Pilar Pozos-Parra, Efrén Mezura-Montes, Edgar Alfredo Portilla-Flores, Eduardo Vega-Alvarado, Maria Bárbara Calva-Yáñez

**Affiliations:** ^1^División Académica de Informática y Sistemas, Universidad Juárez Autónoma de Tabasco, 86690 Cunduacán, TAB, Mexico; ^2^Centro de Investigación en Inteligencia Artificial, Universidad Veracruzana, Sebastián Camacho 5, Centro, 91000 Xalapa, VER, Mexico; ^3^Instituto Politécnico Nacional (IPN-CIDETEC), U. Adolfo López Mateos, 07700 Ciudad de México, DF, Mexico

## Abstract

This paper presents two-swim operators to be added to the chemotaxis process of the modified bacterial foraging optimization algorithm to solve three instances of the synthesis of four-bar planar mechanisms. One swim favors exploration while the second one promotes fine movements in the neighborhood of each bacterium. The combined effect of the new operators looks to increase the production of better solutions during the search. As a consequence, the ability of the algorithm to escape from local optimum solutions is enhanced. The algorithm is tested through four experiments and its results are compared against two BFOA-based algorithms and also against a differential evolution algorithm designed for mechanical design problems. The overall results indicate that the proposed algorithm outperforms other BFOA-based approaches and finds highly competitive mechanisms, with a single set of parameter values and with less evaluations in the first synthesis problem, with respect to those mechanisms obtained by the differential evolution algorithm, which needed a parameter fine-tuning process for each optimization problem.

## 1. Introduction

Nature-inspired algorithms (NIAs) have been successfully used to solve Constrained Numerical Optimization Problems (CNOPs) using constraint-handling techniques [1] given that, originally, these algorithms were designed to deal solely with unconstrained search spaces. NIAs can be comprised of two groups: (1) Evolutionary Algorithms (EAs) [2] based on emulating the process of natural evolution and survival of the fittest and (2) Swarm Intelligence Algorithms (SIAs) [3] based on cooperative behaviors of simple organisms such as insects, birds, fish, or bacteria. EAs are one of the most used metaheuristics. However, SIAs have been gaining popularity among researchers and practitioners, mainly with the Particle Swarm Optimization (PSO) [4] and the Ant Colony Optimization (ACO) [5] algorithms.

Without loss of generality, a CNOP can be defined as

(1) Minimize fx→subject  to: gix→≤0,i=1,…,m hjx→=0,j=1,…,p,

where $\vec{x}=[x_{1},x_{2},\ldots ,x_{n}]\in R^{n}$ is the solution vector and each decision variable *x*_*k*_, *k* = 1,…, *n* is bounded by lower and upper limits *L*_*k*_ ≤ *x*_*k*_ ≤ *U*_*k*_, which define the search space *S*; *m* is the number of inequality constraints and *p* is the number of equality constraints (in both cases, the constraints can be linear or nonlinear). If *F* denotes the feasible region, then it must be clear that *F*⊆*S*. As it is commonly found in the specialized literature of nature-inspired algorithms to solve CNOPs [1, 6, 7] equality constraints are transformed into inequality constraints by using a small tolerance *ɛ* > 0 as follows: $|h_{j}(\vec{x})|-ɛ\leq 0$ , *j* = 1,…, *p*.

In the context of mechanical engineering, synthesis is the design process of mechanical systems [8]. Four-bar mechanisms are widely used in machinery design, since they are the simplest articulated mechanisms for controlled movement with one degree of freedom. The synthesis of these mechanisms is a well-known CNOP, and originally two classical approaches were used for this synthesis: graphical and analytical methods. However, implementing such solutions is a complicated issue and their results are quite limited; for this reason, the design of these mechanisms is a case of hard numerical optimization. There are several types of syntheses; this work addresses the dimensional design of a mechanism, that is, to calculate the length of the necessary links for generating a specific movement [9].

The synthesis of four-bar mechanisms has been carried out with different nature-inspired metaheuristics. In [10] a modified Genetic Algorithm (GA) with a penalty function as constraint-handler was proposed. differential evolution (DE) and a variable control method for deviations were applied in [11]. In [12] the synthesis of a mechanism for tracking a trajectory of *n* points, based on Simulated Annealing (SA), was developed. Regarding this same tracking problem, a performance comparison among three different metaheuristics, GA, DE, and PSO, was presented in [13], while the Artificial Bee Colony (ABC), PSO, a binary GA (BGA), and a hybrid GA-PSO approach were used in [14]. Finally, in [15] the synthesis of a planar four-bar mechanism for position control using the Harmony Search (HS) algorithm was carried out. From the abovementioned literature review, the diversity of nature-inspired algorithms to solve the synthesis of four-bar mechanisms is noticeable.

On the other hand, there are algorithms whose usage in this type of optimization problems has been less explored, as it is the case of the Bacterial Foraging Optimization Algorithm (BFOA), which is a SIA proposed by Passino in 2002 to solve unconstrained numerical optimization problems [16]. BFOA emulates the behavior of bacterium* E. coli* in the search of nutrients in its environment. The goal of each bacterium is to maximize the energy it obtains per each unit of time spent on the foraging process while avoiding noxious substances. BFOA is considered a SIA because bacteria can communicate among them. Such behavior can be summarized in four processes: (1) chemotaxis (swim and tumble movements are performed), (2) swarming (bacteria can communicate with each other to direct their search for nutrients), (3) reproduction (the best bacteria are duplicated and these replaced other bacteria), and (4) elimination-dispersal (the worst bacteria are eliminated and new bacteria are randomly dispersed).

Regarding unconstrained optimization, BFOA has been combined with other algorithms, particularly with EAs, to improve its performance, for example, with a GA in [17–19] and DE in [20]. Mutation operators have been added to BFOA in [21]. Moreover, hybrids with other SIAs [22], and particularly with PSO, are found in the specialized literature [23, 24]. Furthermore, BFOA was also combined with artificial immune system's clonal selection and fuzzy logic within its chemotaxis process in [25].

When dealing with constrained search spaces there are different approaches [26]; for example, in [27], BFOA was adapted to solve CNOPs in a proposal called Modified-BFOA (MBFOA) which used a set of feasibility rules [28] as constraint-handler. Moreover, MBFOA was simplified and improved in its step size handling in the swim operator in [29]. Further modifications were proposed in [30, 31] to solve multiobjective CNOPs, where some four-bar mechanisms were tackled. More recently, MBFOA was further improved to solve CNOPs in the so-called Improved MBFOA (IMBFOA for short) [32] where the idea of two-swim movements within the chemotaxis process was initially explored. However, IMBFOA heavily depends on a local search based on sequential quadratic programming. IMBFOA is the starting point of this research, focused on the optimal synthesis of four-bar mechanisms. Finally, BFOA has been combined with two NIAs BFOA-DE-PSO in [33, 34], and the idea of using micropopulations was explored in [35].

From the above literature review, it was found that NIAs are a valid option to solve four-bar mechanisms. However, the usage of BFOA-based approaches is still scarce. Furthermore, the incorporation of additional variation operators in those BFOA-based approaches is more frequent in unconstrained optimization. The abovementioned is the main motivation of this work, where two improved swims (and different from those in IMBFOA) are proposed to enhance the capabilities of MBFOA to deal with four-bar mechanisms optimization. Furthermore, such two-swim mechanism performance allows eliminating the second-order local search operator. Therefore, the contribution of this work consists in getting knowledge about the type of operators which provide better results in those constrained search spaces defined by the four-bar mechanisms tackled in this research.

The proposed approach is compared against those BFOA-based approaches for constrained optimization (IMBFOA and MBFOA) and also against a DE-based approach designed to solve mechanical design problems. To the best of the authors' knowledge, this is the first time that the variation operators of a BFOA-based approach are studied in such a way that they improve the capabilities of the algorithm to solve a set of four-bar synthesis problems.

The document is organized as follows: in Section 2 the general synthesis of a four-bar mechanism is explained, including the kinematics of the mechanism and its coupler, as well as the specifications of the three case studies to solve. In Section 3, brief descriptions of MBFOA and IMBFOA are presented and the new proposal “Two-Swim MBFOA” (TS-MBFOA) is introduced.  Section 4 shows the results obtained by TS-MBFOA and their comparison against those obtained by other NIAs. Finally, Section 5 presents the conclusions and future work of this research.

## 2. Synthesis of Four-Bar Mechanisms

**Figure 1:**
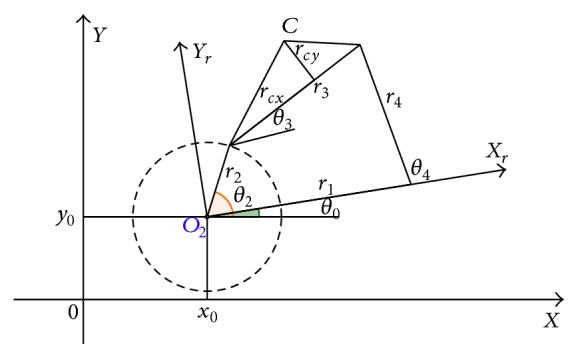
Four-bar mechanism.

Figure 1 shows a planar four-bar mechanism formed by a reference bar *r*_1_, an input bar *r*_2_ (crank), a coupler *r*_3_, and an output bar *r*_4_ (rocker). In order to analyze this mechanism two coordinate systems are established: a system that is fixed to the real world (*OXY*) and another for self-reference (*OX*_*r*_ *Y*_*r*_). (*x*_0_, *y*_0_) is the distance between the origin points of both systems, *θ*_0_ is the rotation angle of the reference system, and *θ*_*i*_  (*i* = 2,3, 4) corresponds to the angle for every bar in the mechanism; finally, the coordinate pair (*r*_*cx*_, *r*_*cy*_) determines the position *C* of the coupler.

### 2.1. Kinematics of the Mechanism

The kinematics of four-bar mechanisms have been extensively treated; a detailed explanation is found in [36, 37]. For analyzing the mechanism position, the closed loop equation can be established as follows:

(2) $$\begin{array}{c} \vec{r_{1}}+\vec{r_{4}}\;\;=\;\;\vec{r_{2}}+\vec{r_{3}}. \end{array}$$

Applying polar notation to each term of (2),

(3) $$\begin{array}{c} r_{1}e^{j\theta_{1}}+r_{4}e^{j\theta_{4}}=r_{2}e^{j\theta_{2}}+r_{3}e^{j\theta_{3}}. \end{array}$$

Using the equation of Euler on (3) and separating the real and imaginary parts,

(4) r1cos⁡θ1+r4cos⁡θ4=r2cos⁡θ2+r3cos⁡θ3,r1sin⁡θ1+r4sin⁡θ4=r2sin⁡θ2+r3sin⁡θ3.

Expressing the equation system (4) in terms of *θ*_4_,

(5) r4cos⁡θ4=r2cos⁡θ2+r3cos⁡θ3−r1cos⁡θ1,r4sin⁡θ4=r2sin⁡θ2+r3sin⁡θ3−r1sin⁡θ1.

The compact form of Freudenstein's equation is obtained by squaring system (5) and adding its terms as follows:

(6) $$\begin{array}{c} A_{1}\cos\theta_{3}+B_{1}\sin\theta_{3}+C_{1}=0, \end{array}$$

where

(7) A1=2r3r2cos⁡θ2−r1cos⁡θ1,B1=2r3r2sin⁡θ2−r1sin⁡θ1,C1=r12+r22+r32−r42−2r1r2cos⁡θ1−θ2.

Then the angle *θ*_3_ can be calculated as a function of the parameters *A*_1_, *B*_1_, *C*_1_, and *θ*_2_; this solution is generated by expressing sin⁡*θ*_3_ and cos⁡*θ*_3_ in terms of tan⁡(*θ*_3_/2):

(8) sin⁡θ3=2tan⁡θ3/21+tan2⁡θ3/2,cos⁡θ3=1−tan2⁡θ3/21+tan2⁡θ3/2.

A second-order lineal equation is obtained by substitution on (6):

(9) C1−A1tan2⁡θ32+2B1tan⁡θ32+A1+C1=0.

From the solution of (9), the angular position *θ*_3_ is given by (10):

(10) $$\begin{array}{c} \theta_{3}=2\arctan\left[\;\frac{-B_{1}\pm \sqrt{B_{1}^{2}+A_{1}^{2}-C_{1}^{2}}}{C_{1}-A_{1}}\;\right]. \end{array}$$

A similar process is carried out to get *θ*_4_ from (4) using Freudenstein's equation. The correct sign for the radical must be selected in the equations for *θ*_3_ and *θ*_4_, according to the configuration of the mechanism.  Table 1 indicates the signs related with both configurations.

### 2.2. Kinematics of the Coupler

Since the point of interest in the coupler is *C*, to determine its position in the reference system *OX*_*r*_ *Y*_*r*_ it has to be established that

(11) Cxr=r2cos⁡θ2+rcxcos⁡θ3−rcysin⁡θ3,Cyr=r2sin⁡θ2+rcxsin⁡θ3+rcycos⁡θ3.

In the global coordinate system, this point is expressed as

(12) $$\begin{array}{c} \left[\begin{array}{c} C_{x}\\ C_{y} \end{array}\right]=\left[\begin{array}{cc} \cos\theta_{0} & -\sin\theta_{0}\\ \sin\theta_{0} & \cos\theta_{0} \end{array}\right]\left[\begin{array}{c} C_{xr}\\ C_{yr} \end{array}\right]+\left[\begin{array}{c} x_{0}\\ y_{0} \end{array}\right]. \end{array}$$

Equations (11) and (12) and the expressions from the kinematics of the mechanism are sufficient to calculate the position of *C* along the trajectory.

### 2.3. Design Constraints

One of the most important aspects involved in a mechanism design is to accomplish the constraints on its performance, which are related to mobility criteria and the size and shape of the mechanism itself.

#### 2.3.1. Grashof's Law

Grashof's law is a fundamental consideration when designing a four-bar mechanism, since it defines the criteria to ensure complete mobility for at least one link of that mechanism. This law establishes that* for a planar four-bar linkage, the sum of the shortest and the largest bars cannot be larger than the sum of the remaining bars, if a continual relative rotation between two elements is desired* [8]. If *s* is the length of the shortest link, *l* represents the largest bar, and *p*, *q* indicate the remaining elements, it is established that

(13) $$\begin{array}{c} l+s\leq p+q. \end{array}$$

In this work, Grashof's law is given by

(14) $$\begin{array}{c} r_{1}+r_{2}\leq r_{3}+r_{4}. \end{array}$$

Therefore, to ensure that the solution method fulfills this law, the following constraints were established:

(15) r2<r3,r3<r4,r4<r1.

#### 2.3.2. Sequence of Input Angles

Since the general problem of synthesis addressed in this work is the generation of trajectories based on sequences of successive precision points representing different positions of the coupler, the values of the crank angles have to be ordered in correspondence with these sequences. If the angle for a specific point *i* is denoted as *θ*_2_^*i*^, it is required that

(16) $$\begin{array}{c} \theta_{2}^{1}<\theta_{2}^{2}<\cdots <\theta_{2}^{K}, \end{array}$$

where *K* is the number of precision points.

### 2.4. Optimization Strategies

After properly establishing the kinematics of the mechanism, the design problem can be defined as a numerical optimization case, and then it is necessary to specify the appropriate mathematical expressions for evaluating the performance of the system.

#### 2.4.1. Objective Function

This work addresses the synthesis of a planar mechanism in order to calculate the length of its bars, the rotation angle in respect to the reference system, the distance between the coordinate systems, and the set of angles for the input bar to generate a trajectory corresponding to a sequence of precision points. In the global coordinate system *OXY*, the point of the precision pair *C*_*d*_^*i*^ is indicated as

(17) $$\begin{array}{c} C_{d}^{i}={\left[\;C_{xd}^{i},C_{yd}^{i}\;\right]}^{T}. \end{array}$$

The set of *K* pairs of precision points is defined as

(18) $$\begin{array}{c} \Omega =\left\{\;C_{d}^{i}\;|\;i\in K\;\right\}. \end{array}$$

Then, given a set of values of the mechanism bars and their parameters *x*_0_, *y*_0_, *θ*_0_, each point of the coupler can be expressed as a function of the input bar position:

(19) $$\begin{array}{c} C^{i}={\left[\;C_{x}\left(\;\theta_{2}^{i}\;\right),C_{y}\left(\;\theta_{2}^{i}\;\right)\;\right]}^{T}. \end{array}$$

Accordingly, it is desired to minimize the distance (error) between the precision point *C*_*d*_^*i*^ and the calculated point *C* ^*i*^. To quantify the overall error the following function is proposed:

(20) $$\begin{array}{c} \text{error}=\overset{K}{\underset{i=1}{\sum }}\left[\;{\left(\;C_{xd}^{i}-C_{x}^{i}\;\right)}^{2}+{\left(\;C_{yd}^{i}-C_{y}^{i}\;\right)}^{2}\;\right]. \end{array}$$

#### 2.4.2. Case Studies

(*1) M01*. It is the design of a four-bar mechanism that follows a linear vertical path defined by a sequence of six precision points, without a previously established synchronization. The set of precision points is defined as

(21) Ω=20,20,20,25,20,30,20,35,20,40,20,45.

The vector of design variables is

(22) p→=p1,p2,p3,p4,p5,p6,p7,p8,p9,p10,p11,p12,p13,p14,p15,

where

(23) p→=r1,r2,r3,r4,rcx,rcy,θ0,x0,y0,θ21,θ22,θ23,θ24,θ25,θ26.

The first four variables correspond to the lengths of the bars in the mechanism presented in Figure 1, the following two are the position of the coupler, *θ*_0_ is the orientation angle of the system with respect to the horizontal, *O*_2_ = (*x*_0_, *y*_0_) is its coordinate position, and the last six are the angle values for the input bar *r*_2_. The boundaries for each design variable are defined as

(24) p1,p2,p3,p4∈0,60,p5,p6,p8,p9∈−60,60,p7,p10,p11,p12,p13,p14,p15∈0,2π.

The single-objective numerical optimization problem for this case is described by the following:

(25) min⁡ fp→=∑i=1NCxdi−Cxi2+Cydi−Cyi2,p∈R15subject  to: g1p→=p1+p2−p3−p4≤0, g2p→=p2−p3≤0, g3p→=p3−p4≤0, g4p→=p4−p1≤0, g5p→=p10−p11≤0, g6p→=p11−p12≤0, g7p→=p12−p13≤0, g8p→=p13−p14≤0, g9p→=p14−p15≤0.

(*2) M02*. It is the design of a four-bar mechanism that follows a trajectory defined by a sequence of five unaligned precision points, with a previously established synchronization for each point. The set of precision points is defined as

(26) Ω=3,3,2.759,3.363,2.372,3.663,1.89,3.862,1.355,3.943.

For this case it is considered that *x*_0_, *y*_0_, *θ*_0_ = 0. The restriction given by (16) is not considered since the sequence of input angles is set by

(27) $$\begin{array}{c} \theta_{2}^{i}=\left\{\;\frac{2\pi }{12},\frac{3\pi }{12},\frac{4\pi }{12},\frac{5\pi }{12},\frac{6\pi }{12}\;\right\}. \end{array}$$

The vector of design variables is

(28) $$\begin{array}{c} \vec{p}=\left\{\;p_{1},p_{2},p_{3},p_{4},p_{5},p_{6}\;\right\}, \end{array}$$

where

(29) $$\begin{array}{c} \vec{p}=\left\{\;r_{1},r_{2},r_{3},r_{4},r_{cx},r_{cy}\;\right\}. \end{array}$$

The upper and lower values for the design variables are defined as

(30) p1,p2,p3,p4∈0,50,p5,p6∈−50,50.

The objective function is defined by the following:

(31)min⁡ fp→=∑i=1NCxdi−Cxi2+Cydi−Cyi2,p∈R6(32)subject  to: g1p→=p1+p2−p3−p4≤0, g2p→=p2−p3≤0, g3p→=p3−p4≤0, g4p→=p4−p1≤0.

(*3) M03*. It is the design of a four-bar mechanism for tracking a trajectory delimited by pairs of precision points. This case considers a sequence with ten pairs of precision points given by the coordinates shown in Table 2.

The vector of design is

(33) p→=p1,p2,p3,p4,p5,p6,p7,p8,p9,p10,p11,p12,p13,p14,p15,p16,p17,p18,p19,

where

(34) $$\begin{array}{c} \vec{p}=\left\{\;r_{1},r_{2},r_{3},r_{4},r_{cx},r_{cy},\theta_{0},x_{0},y_{0},\theta_{2}^{1},\ldots ,\theta_{2}^{10}\;\right\} \end{array}$$

and its variables are limited by

(35) p1,p2,p3,p4∈0,60,p5,p6,p8,p9∈−60,60,p7,p10⋯p19∈0,2π.

Because in this case the trajectory is defined by pairs of precision points, the objective function in (20) is modified in order to consider the error with respect to each point. Therefore, the new function is given by the following:

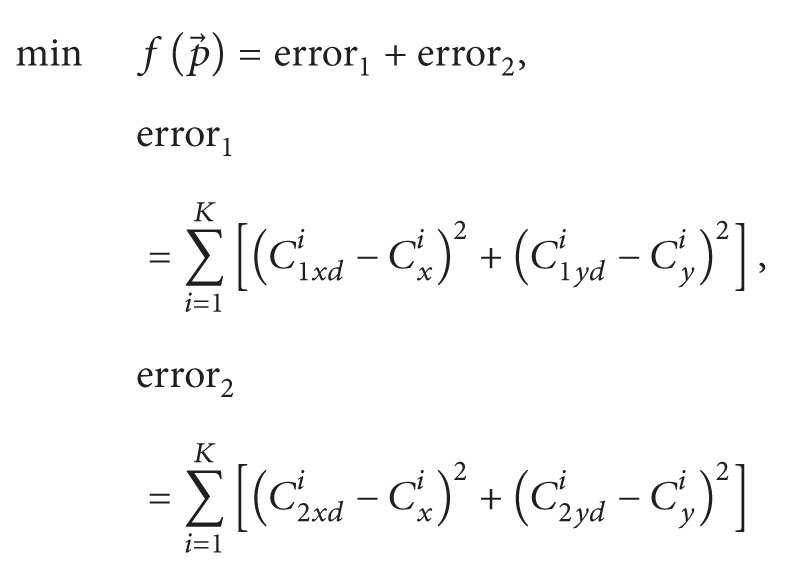

(36)

(37) subject  to: g1p→=p1+p2−p3−p4≤0, g2p→=p2−p3≤0, g3p→=p3−p4≤0, g4p→=p4−p1≤0, g5p→=p10−p11≤0, g6p→=p11−p12≤0, g7p→=p12−p13≤0, g8p→=p13−p14≤0, g9p→=p14−p15≤0, g10p→=p15−p16≤0, g11p→=p16−p17≤0, g12p→=p17−p18≤0, g13p→=p18−p19≤0.

Finally, it is important to note that the complexity of the study cases presented in the paper is high, due to two aspects. (1) A large number of precision points that must touch the mechanism: in the state of the art of synthesis mechanisms, cases with four precision points maximum are solved using graphic methods or MPM's. (2) Values of design variables *θ*_*i*_^2^ with *i* = 1,…, 6 for case *M*01 and *i* = 1,…, 10 for case *M*02: these must have an ascending or descending order, which implies a strong constraint for finding solution vectors to ensure a proper mechanism functioning. Additionally, cases *M*01 and *M*02 have not previously undergone a synchronization on the mechanism input bar.

## 3. Two-Swim Modified Bacterial Foraging Optimization Algorithm (TS-MBFOA)

TS-MBFOA is inspired by the ideas of IMBFOA, a recently proposed BFOA-based algorithm to solve CNOPs by using two-swim operators, a skew mechanism for the initial swarm of bacteria, a second-order local search operator, and a limited usage of the reproduction step [32]. To get a self-contained paper, in the next subsections, MBFOA, IMBFOA's base algorithm, is presented. After that, IMBFOA is detailed. Finally, TS-MBFOA is introduced.

### 3.1. Modified Bacterial Foraging Optimization Algorithm (MBFOA)

MBFOA is based on the original BFOA [16], but it was proposed to solve CNOPs. Each one of its elements is detailed as follows.

(i) A bacterium *i* represents a potential solution to the CNOP (i.e., a *n*-dimensional real-value vector identified as $\vec{x}$ in Section 1), and it is denoted as *θ* ^*i*^(*j*, *G*), where *j* is its chemotaxis loop index and *G* is a generational (cycle) loop index. Within a cycle, three inner processes are carried out: chemotaxis, reproduction, and elimination-dispersal. Swarming process is added to the chemotaxis process.
(ii) *Chemotaxis*. In this process, each bacterium in the current swarm performs a tumble-swim movement. The tumble, as proposed by Passino [16], consists of a search direction *ϕ*(*i*) generated at random with uniform distribution as presented in the following:
(38) $$\begin{array}{c} \phi \left(\;i\;\right)=\frac{\Delta \left(i\right)}{\sqrt{\Delta {\left(i\right)}^{T}\Delta \left(i\right)}}, \end{array}$$
 where Δ(*i*) is a *n*-dimensional real-value vector generated at random with uniform distribution where each one of its elements has values between [−1,1].
 The swim allows the bacterium *θ* ^*i*^(*j*, *G*) to follow the search direction and move to a new position *θ* ^*i*^(*j* + 1, *G*). The swim is computed as indicated in the following:
(39) $$\begin{array}{c} \theta^{i}\left(\;j+1,G\;\right)=\theta^{i}\left(\;j,G\;\right)+C\left(\;i\;\right)\phi \left(\;i\;\right), \end{array}$$
 where *C*(*i*) is the step size vector and its values are calculated by considering the limits of each design variable *k*, defined by the expression in the following:
(40) $$\begin{array}{c} C{\left(\;i\;\right)}_{k}=R*\left(\;\frac{\Delta x_{k}}{\sqrt{n}}\;\right),\;k=1,\ldots ,n, \end{array}$$
 where Δ*x*_*k*_ is the difference between the upper and lower limits of each variable *x*_*k*_: *U*_*k*_ − *L*_*k*_, *n* is the number of variables, and *R* ∈ [0,1] is a user-defined parameter that scales the step size value of bacteria. This vector remains fixed during the search process. MBFOA, like other BFOA-based algorithms as shown in [26], is particularly sensitive to this parameter and such behavior has motivated further studies as that in [29].
 If the new position, *θ* ^*i*^(*j* + 1, *G*), is better with respect to the previous position *θ* ^*i*^(*j*, *G*) [28] (i.e., (1) both positions are feasible, but the new position has a better objective function value, (2) the new position is feasible while the previous one is not, or (3) both positions are infeasible, but the new position has a lower sum of constraint violation), another swim in the same direction will be carried out by taking this better solution as the new starting position. Otherwise, a new tumble is computed. The process stops after *N*_*c*_ attempts (parameter defined by the user).
(iii) *Swarming*. MBFOA includes an attractor movement within the chemotaxis process, which lets each bacterium in the swarm follow the bacterium located in the most promising region of the search space, that is, either the feasible bacterium with the best objective function value or the bacterium with the lowest sum of constraint violation if no feasible bacteria are found in the current swarm. Such information is given by the three feasibility rules used in the chemotaxis process. The movement is detailed in the following:
(41) $$\begin{array}{c} \theta^{i}\left(\;j+1,G\;\right)=\theta^{i}\left(\;j,G\;\right)+\beta \left(\;\theta^{B}\left(\;G\;\right)-\theta^{i}\left(\;j,G\;\right)\;\right), \end{array}$$
 where *θ* ^*i*^(*j* + 1, *G*) is the new position of bacterium *i*, *θ* ^*i*^(*j*, *G*) is the current position of bacterium *i*, *θ* ^*B*^(*G*) is the current position of the best bacterium in the swarm so far at cycle *G*, and *β* (user-defined parameter) defines the closeness of the new position of bacterium *i* with respect to the position of the best bacterium *θ* ^*B*^(*G*). The attractor movement is applied once within the chemotaxis loop. In the remaining steps, the tumble-swim movement is used. Both the tumble-swim and swarming movements can generate variable values outside their limits. Therefore, a simple repair mechanism is used as in [38], where the violated value is multiplied by 2 and the violated limit is subtracted (i.e., *x*_valid_ = 2*∗*violated_limit − *x*_invalid_).
(iv) *Reproduction*. The swarm is sorted based on the same three rules adopted in the chemotaxis process and the first *S*_*r*_ are cloned (these bacteria are considered as the best ones), and the remaining *S*_*b*_ − *S*_*r*_ (the worst bacteria) are eliminated (*S*_*b*_ is the swarm size).
(v) *Elimination-Dispersal*. This process eliminates only the worst bacterium *θ* ^*w*^(*j*, *G*) based on the already mentioned feasibility rules, and a new randomly generated bacterium is inserted as a replacement.

In Algorithm 1 the corresponding MBFOA pseudocode is presented, and its user-defined parameters are summarized in the caption.

### 3.2. Improved MBFOA (IMBFOA)

IMBFOA was designed to improve MBFOA in its performance to solve CNOPs. Four changes were promoted: (1) two-swim movements within the chemotaxis process, one for exploration and another one for exploitation, (2) a skew mechanism for the initial swarm of bacteria, (3) a local search operator based on sequential quadratic programming, and (4) a reduction on the usage of the reproduction process. Below is the description of each change.

(i) *Two-Swim Operators*. In the chemotaxis process, instead of the [−1,1] interval, the range for the tumble was set to [*υ*, *τ*], where *υ* and *τ* are user-defined parameters, −1 ≤ *υ* < 0, 0 < *τ* ≤ 1.
 The first swim, focused on exploration, is computed as indicated in the following:
(42) $$\begin{array}{c} \theta^{i}\left(\;j+1,G\;\right)=\theta^{i}\left(\;j,G\;\right)+\phi \left(\;i\;\right), \end{array}$$
 where *ϕ*(*i*) is computed as in (38), but now considering the updated range and not using the step size vector. The second swim, focused on exploitation, is computed as indicated in the following:
(43) $$\begin{array}{c} \theta^{i}\left(\;j+1,G\;\right)=\theta^{i}\left(\;j,G\;\right)+C\left(\;i,G\;\right)\phi \left(\;i\;\right), \end{array}$$
 where *C*(*i*, *G*) is a dynamic step size vector [29]. However, each value *k* of vector *C*(*i*, *G*) decreases dynamically at each cycle of the algorithm as indicated in the following:
(44) $$\begin{array}{c} C{\left(\;i,G+1\;\right)}_{k}=C{\left(\;i,G\;\right)}_{k}\frac{G}{GMAX},\;k=1,\ldots ,n, \end{array}$$
 where *C*(*i*, *G* + 1)_*k*_ is the new step size value for variable *k*, while *G* and *G*MAX are the current and maximum number of cycles of the algorithm, respectively. The initial *C*(*i*, 0) is computed as indicated in (40), but the *R* parameter is no longer used.
 The first swim is applied until no improvement is obtained, and then the second swim takes place and so on. The process stops, as in the chemotaxis process in MBFOA, after *N*_*c*_ attempts.
(ii) *Skew Mechanism for the Initial Swarm*. The initial swarm of bacteria *S*_*b*_ is generated by considering three groups. In the first group there are randomly generated bacteria but with their location skewed to the lower limit of the decision variables *L*_*k*_. In the second group there are randomly generated bacteria but with their location skewed to the upper limit of the decision variables *U*_*k*_. Finally, a third group of randomly generated bacteria are created as in the original MBFOA (i.e., without any skew). The three groups use random values with uniform distribution. The details to set the limits per variable for the first and second group are presented in the following:
(45) $$\begin{array}{c} \left[\;L_{k},L_{k}+\left(\;\frac{\left(U_{k}-L_{k}\right)}{ss}\;\right)\;\right],\\ \left[\;U_{k}-\left(\;\frac{\left(U_{k}-L_{k}\right)}{ss}\;\right),U_{i}\;\right], \end{array}$$
 where ss is the skew size. A high value decreases the skew effect, while a low value increases it.
(iii) *Local Search Operator*. Sequential Quadratic Programming (SQP) [39] is the local search operator in IMBFOA. This search is applied to the best bacterium in the swarm after the chemotaxis, swarming, reproduction, and elimination-dispersal processes. The user can define the local search operator usage frequency with the LS_*G*_ parameter.
(iv) *Scarce Usage of the Reproduction Step*. To reduce premature convergence due to bacteria duplication, the reproduction takes place only at certain cycles of the algorithm, defined by the RepCycle parameter.

Algorithm 2 includes the IMBFOA pseudocode and its parameters are in the caption.

### 3.3. Two-Swim MBFOA (TS-MBFOA)

TS-MBFOA revisits the two swims originally proposed in IMBFOA to enhance its search capabilities and to simplify them as well. In this way, two new swims to be applied within the chemotaxis process are proposed in this work. The first one of them aims to complement the swarming operator by letting a bacterium to explore other areas of the search space with the guide of randomly chosen bacteria. The second swim focuses on slight movements of the bacterium in its vicinity by using the original swim proposed by Passino [16], but with very small step size values.

The details of each one of the two proposed swims are presented below.

(*1) Exploration Swim*. The first swim is computed as indicated in the following:

(46) θij+1,G=θij,G+β−1θr1j,G−θr2j,G,

where *β* is the user-defined parameter utilized in MBFOA's swarming operator and its value is now greater than 1. *θ* ^*r*_1_^(*j*, *G*) and *θ* ^*r*_2_^(*j*, *G*) are two bacteria randomly selected from the swarm (*i* ≠ *r*_1_ ≠ *r*_2_). This swim operator uses the position of such two bacteria to determine a search direction considering the current position of the bacterium ready to swim *θ* ^*i*^(*j*, *G*) as the starting point.

**Figure 2:**
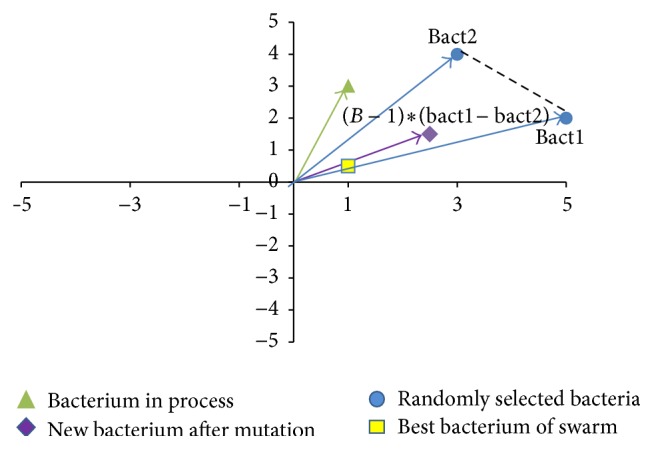
Graphical example of the first exploration swim.

Figure 2 shows the behavior of this swim operator using a space of two decision variables, each one into a range of [−5,5]. In this example, the new position of the bacterium after the swim will fall in the purple spot defined by bact1 and bact2, which are *θ*_1_^*r*^(*j*, *G*) and *θ*_2_^*r*^(*j*, *G*), respectively. The best bacterium is included so as to remark that this operator aims to find different regions of the search space (i.e., not those on the neighborhood of the best current solution as the swarming movement promotes).

(*2) Exploitation Swim*. The second swim returns to be original swim based on random search directions but is now coupled with small random step size values to precisely favor fine movements, as indicated in the following:

(47) $$\begin{array}{c} \theta^{i}\left(\;j+1,G\;\right)=\theta^{i}\left(\;j,G\;\right)+C\left(\;i,G\;\right)\phi \left(\;i\;\right), \end{array}$$

where the step size values comprise a *n*-dimensional random vector called again *C*(*i*, *G*) [29], calculated at each generation as shown in the following:

(48) $$\begin{array}{c} C{\left(\;i,G\;\right)}_{k}=R*\Delta_{\left(i_{k}\right)},\;k=1,\ldots ,n, \end{array}$$

where Δ_(*i*_*k*_)_ is a randomly generated value with uniform distribution within [*L*_*k*_, *U*_*k*_] of decision variable *k*. *R* is a user-defined parameter to scale the step size, and its value should be close to zero, for example, 5.00*E* − 03. At the first cycle, the step size is calculated using just Δ_(*i*_*k*_)_ to allow bacteria in the initial swarm to move in different directions within the search space while avoiding attractors at the start of the process, as suggested in [40].

**Figure 3:**
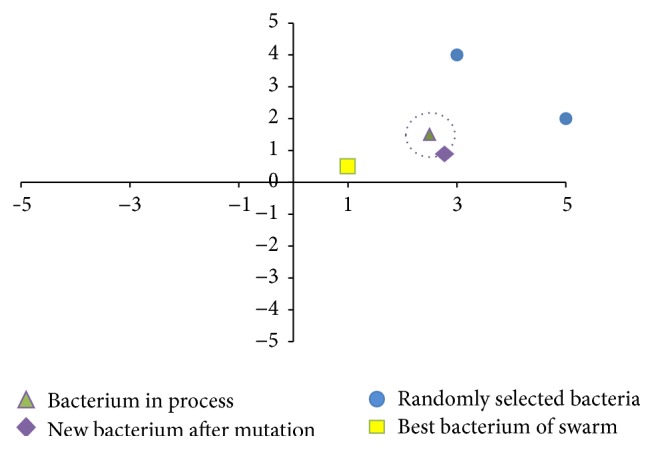
Random swim behavior.

Figure 3 shows the swim behavior, where the bacterium represented as a green triangle will move by using a random search direction but close to its current position, regardless of the positions of other bacteria in the swarm.

Algorithm 3 presents TS-MBFOA pseudocode, and its parameters are detailed in the caption.

The combined expected effect of both proposed swims with the swarming operator, all three inside the chemotaxis process, is an enhanced ability to avoid local optimum solutions and a promotion of a faster convergence. Such effect is possible because of the fact that TS-MBFOA has a swim for exploration (first proposed swim), a swim to favor convergence (swarming operator from MBFOA), and a fine-swim to further improve good quality solutions (second proposed swim).

Finally, it is important to remark that the local search based on SQP is not used in TS-MBFOA. Therefore, second-order information is not required as it was the case with IMBFOA.

## 4. Results and Analysis

TS-MBFOA was used to solve the three four-bar synthesis design problems stated in Section 2. A summary of their main features is presented in Table 3. Four experiments were designed to (1) assess the effectiveness of the proposed swims compared with the swims in IMBFOA and MBFOA, (2) compare the final results of TS-MBFOA against those of IMBFOA and MBFOA, (3) compare the final results of TS-MBFOA now against those obtained by a DE-based approach to solve mechanical engineering problems [41], and (4) simulate the best four-bar systems obtained by each one of the three algorithms in each one of the optimization problems to analyze their behavior from a mechanical point of view. The Wilcoxon Signed-Rank Test (WSRT) [42] was used to validate the differences observed in the samples of 30 independent runs computed per algorithm per test problem in the experiments. TS-MBFOA was coded in MATLAB R2009b and executed on a PC with a 3.5 Core 2 Duo Processor, 4 GB of RAM, and 64-bit Windows 7 operating system.

### 4.1. Performance Measures

To evaluate the behavior of the compared algorithms, the following performance measures for nature-inspired constrained optimization, taken from [43], were computed:

(i) *Feasible run*: a run where at least one feasible solution is found within Max_Evals.
(ii) *Feasible rate* = (number of feasible runs)/total runs.
(iii) *Successful swim*: A swim movement where the new position is better (based on the feasibility rules) than the original position.
(iv) *Successful swim rate* = (number of successful swims)/total swims, where total swims = *S*_*b*_ × *N*_*c*_ × *G*MAX.

### 4.2. Parameter Setting

The parameter setting for nature-inspired algorithms is an open problem [44]. Therefore, to get suitable parameter values for the proposed algorithm, a tuning process was carried out by the iRace tool [45]. iRace implements the iterated racing procedure for automatic algorithm configuration. Iterated racing is a generalization of the iterated F-race and consists of three phases: (1) sampling new parameter configurations with a particular distribution, (2) choosing the most competitive configurations by means of racing, and (3) updating the sampling distribution to favor better configurations. For details about iRace the reader is referred to [45].

The user-defined parameter of TS-MBFOA is shown in Table 4. The parameter values for MBFOA and IMBFOA were taken from [29] and [32], respectively.

The set of parameters for TS-MBFOA in Table 4 is one out of four sets provided by iRace. The other three are the following: (1) *S*_*b*_ = 60, *N*_*c*_ = 12, *R* = 1.89*E* − 2, *S*_*r*_ = 1, *β* = 1.32, RepCycle = 100, ss = 5, (2) *S*_*b*_ = 40, *N*_*c*_ = 22, *R* = 1.50*E* + 0, *S*_*r*_ = 2, *β* = 1.75, RepCycle = 80, ss = 8, and (3) *S*_*b*_ = 40, *N*_*c*_ = 24, *R* = 1.34*E* − 1, *S*_*r*_ = 5, *β* = 1.54, RepCycle = 100, ss = 8. As it can be seen, four parameters in the sets have different values: *N*_*c*_, *R*, *S*_*r*_, and *β*. This suggests that TS-MBFOA is not very sensitive to those parameters. From those four parameters, *R* has been reported as very sensitive in previous MBFOA versions [29]. However, TS-MBFOA shows less sensitivity to its value. On the other hand, the parameters with similar values in the sets are *S*_*b*_, RepCycle, and ss. This suggests that TS-MBFOA requires a more careful tuning of the swarm size, the reproduction frequency, and the initial skew in the population. However, the tuning process could deal with such sensitivity.

### 4.3. Experiment 1: Effectiveness of the Proposed Swims

**Figure 4:**
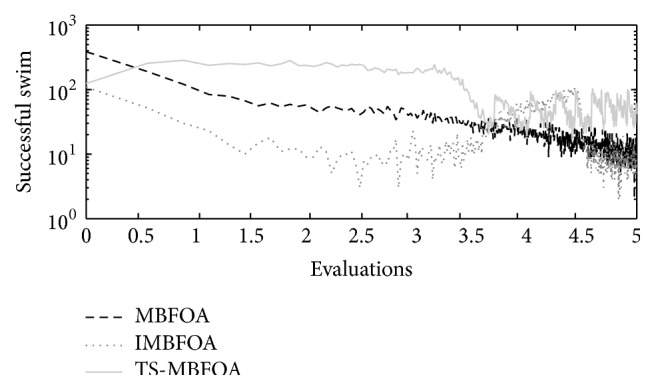
Successful swims by MBFOA, IMBFOA, and TS-MBFOA in *M*01 problem in the execution located in the median value of 30 independent runs.

**Figure 5:**
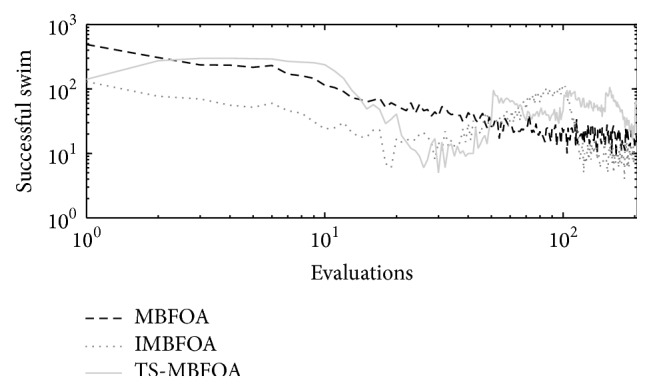
Successful swims by MBFOA, IMBFOA, and TS-MBFOA in *M*02 problem in the execution located in the median value of 30 independent runs.

**Figure 6:**
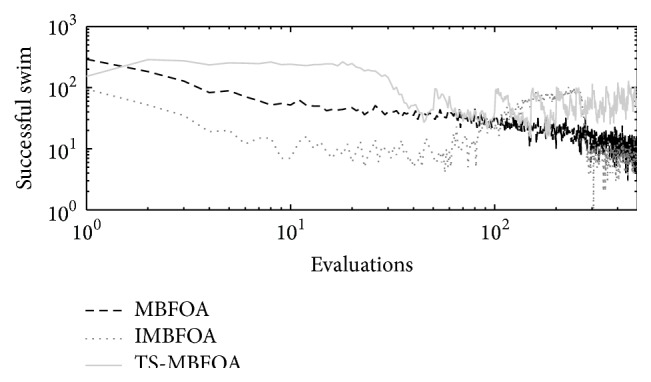
Successful swims by MBFOA, IMBFOA, and TS-MBFOA in *M*03 problem in the execution located in the median value of 30 independent runs.

The number of successful swims per generation obtained by TS-MBFOA, IMBFOA, and MBFOA on the three four-bar synthesis problems (*M*01, *M*02, and *M*03) is presented in Figures 4, 5, and 6, where the run located in the median value of 30 independent runs is plotted. In the three figures, the effectiveness of the two proposed swims included in TS-MBFOA was superior in most of the process and particularly late in the search. On the other hand, the number of successful swims in MBFOA showed a decreasing tendency in the three synthesis problems. Finally, the number of successful swims in IMBFOA was the lowest in the three problems but showed some improvement at the end of the search, but it did not outperform those successful swims by TS-MBFOA. To provide further evidence to the above finding, the successful swim rates in problem *M*01 were 3.68% by MBFOA, 2.16% by IMBFOA, and 7.26% by TS-MBFOA. In problem *M*02 the successful swim rates were 2.31%, 3.76%, and 4.36%, by MBFOA, IMBFOA, and TS-MBFOA, respectively. Finally, in problem *M*03, the rates were 2.14%, 3.76% and 6.53% by MBFOA, IMBFOA, and TS-MBFOA, respectively. As a conclusion of this first experiment, the two proposed swims were able to generate a greater number of better solutions during the search, mainly in late generations, unlike the swims in IMBFOA and BFOA. It remains to be seen if such behavior leads to better final results.

### 4.4. Experiment 2: Final Results Comparison among BFOA-Based Approaches

As a first element of analysis, the feasible rates obtained by TS-MBFOA, IMBFOA, and MBFOA in the three four-bar synthesis problems are shown in Table 5. It is clear that the three algorithms were able to consistently reach the feasible region of the search space.

The statistical results obtained by MBFOA, IMBFOA, and TS-MBFOA on the three four-bar synthesis problems are presented in Table 6 in terms of best, average, and standard deviation values of 30 independent runs. According to the 95%-confidence Wilcoxon Signed-Rank Test, the differences observed in the samples of runs in Table 6 are significant. Based on such information, TS-MBFOA outperformed IMBFOA and BFOA in the three optimization problems.

**Figure 7:**
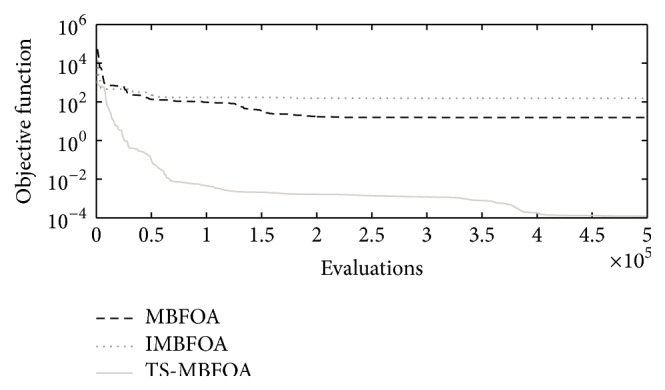
Convergence plots by each BFOA-based algorithm in problem *M*01.

**Figure 8:**
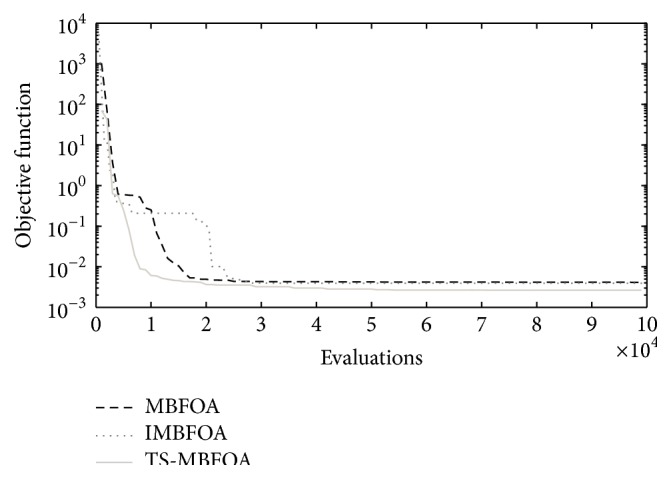
Convergence plots by each BFOA-based algorithm in problem *M*02.

**Figure 9:**
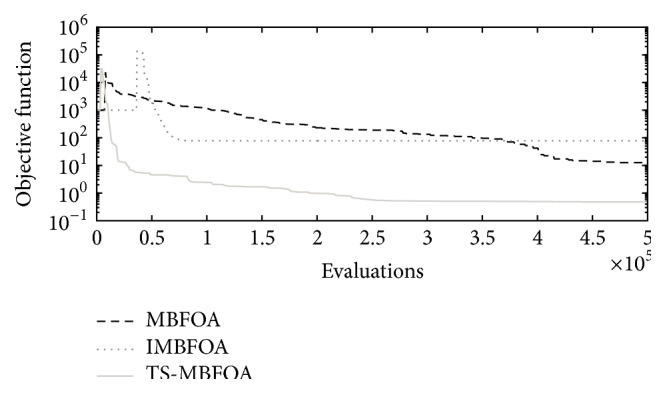
Convergence plots by each BFOA-based algorithm in problem *M*03.

To further understand the behavior of each BFOA-based algorithm, the convergence plots for each optimization problem are shown in Figures 7, 8, and 9 using the run located in the median value of the 30 independent runs. To complement the information, in Table 7, the objective function value of the best solution found in such run is presented per algorithm per optimization problem.

Those results suggest that the combination of the two proposed swims allowed TS-MBFOA to avoid local optimum solutions and find even more promising areas in the feasible region of the search space. In contrast, IMBFOA and MBFOA got trapped in those local attractors. Finally, based on the best solution found in the run located in the median value out of the 30 independent runs, TS-MBFOA was the most consistent algorithm to reach competitive values.

### 4.5. Experiment 3: Comparison between TS-MBFOA and an Evolutionary Algorithm for Mechanical Design

The results of TS-MBFOA were compared against those obtained by a differential-evolution-based approach designed to solve mechanical design problems [41]. The parameter values used for such algorithm were the following: 100 individuals and 7500 generations for problem *M*01, 100 individuals and 1000 generations for problem *M*02, and 100 individuals and 5000 generations for problem *M*03. *F* and *CR* values were randomly generated at each generation within the following intervals: [0.3, 0.9] and [0.8, 1.0], respectively.

Table 8 includes the statistical results of 30 independent runs carried out by TS-MBFOA and the DE-based approach. It is important to mention that in the 30 runs, both algorithms found feasible solutions. Moreover, the 95%-confidence Wilcoxon test indicated that the differences between the algorithms in the final results were not significant.

Despite the fact that no significant differences were observed in the results obtained by TS-MBFOA and the DE-based approach, TS-MBFOA was able to find those competitive results by using a single parameter setting, while the DE-based approach required a fine-tuning for each optimization problem. Furthermore, TS-MBFOA required less evaluations to reach such results in problem *M*01 (500,000 against 750,000 evaluations in problem *M*01).

### 4.6. Experiment 4: Simulation of Best Solutions

**Figure 10:**
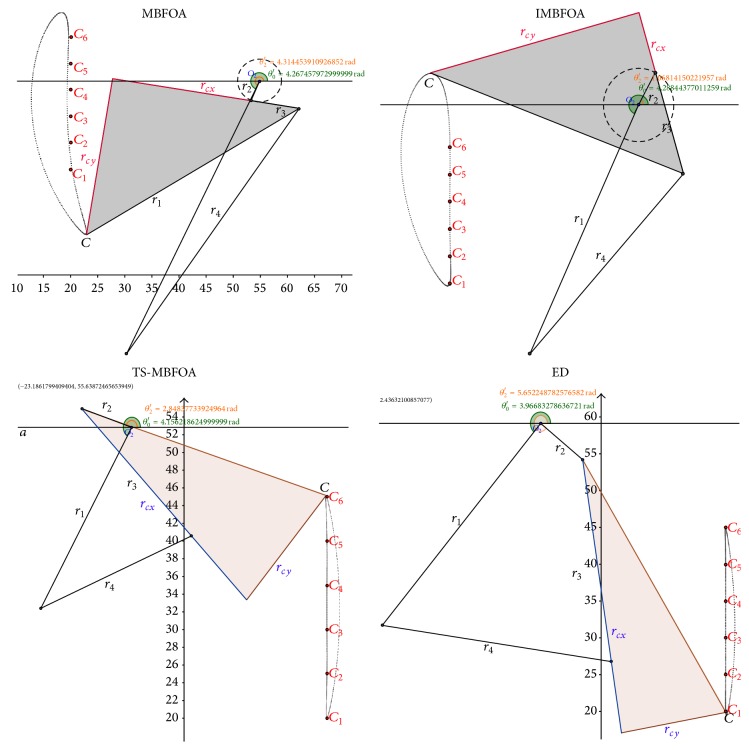
Problem *M*01 best solution simulation.

**Figure 11:**
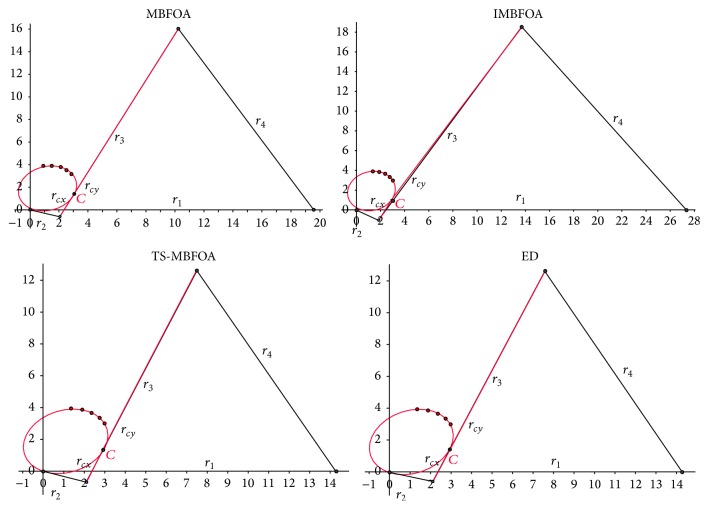
Problem *M*02 best solution simulation.

**Figure 12:**
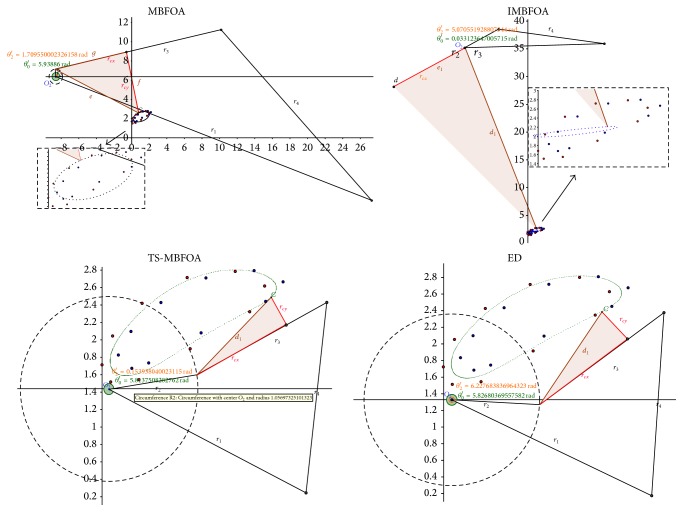
Problem *M*03 best solution simulation.

This final experiment simulated the mechanisms corresponding to the best solutions found by MBFOA, IMBFOA, TS-MBFOA, and the DE-based algorithms for the three problems. The results are analyzed from a mechanical point of view. The decision variable values of each best solution per algorithm per optimization problem are presented in Table 9. The graphical representations of the simulations are shown in Figures 10, 11, and 12 for problems *M*01, *M*02, and *M*03, respectively.

Regarding problem *M*01, Figure 10 indicates that the four algorithms found mechanisms (solutions) whose trajectories pass over the six precision points. However, the mechanisms generated by MBFOA and IMBFOA are less efficient in terms of time and energy consumption because their recovering loops to start tracking the points again are much longer than those obtained by TS-MBFOA and the DE-based approach.

The mechanisms provided by TS-MBFOA, the DE-based approach, and MBFOA in problem *M*02 (Figure 11) were equally good from a mechanical point of view, that is, the trajectories of the three mechanisms pass over the five precision points and their elements vary in less than 25% among them. The exception was the mechanism obtained by IMBFOA because it was deficient; that is, its transmission did not pass over the trajectory specified by the five precision points.

For the most complex problem *M*03 (Figure 12), the mechanisms provided by TS-MBFOA and the DE-based approach showed a similar path through the precision point pairs. Furthermore, the length of the bars is quite uniform (see Table 9). In contrast, the mechanism found by MBFOA fails in some precision point pairs and the length of its bars is not as uniform as those of the mechanisms obtained by TS-MBFOA and the DE-based approach. Large bars may produce undesired effects such as bad alignment, weak balancing because of a transverse flexion produced by internal loads, and a bigger stress between mechanical elements on the tightening points related with the transmission angle of the mechanism. Finally, IMBFOA failed to provide a competitive mechanism based on both number of precision point pairs covered and bar length uniformity.

From this last experiment, the simulation of the best solutions suggests that TS-MBFOA was able to find, from a mechanical point of view, high-quality four-bar mechanisms in the three instances presented in this work. Particularly in problem *M*01, TS-MBFOA was able to find a very competitive solution with less evaluations with respect to the DE-based approach, an algorithm whose performance has been highly competitive when solving mechanical design problems [41].

## 5. Conclusions and Future Work

This work proposed two-swim operators for the modified bacterial foraging optimization algorithm (TS-MBFOA), to solve three instances of the synthesis of four-bar planar mechanisms: (1) design of a four-bar mechanism that follows a linear vertical path defined by a sequence of six precision points, without a previously established synchronization (*M*01), (2) design of a four-bar mechanism that follows a trajectory defined by a sequence of five unaligned precision points, with a previously established synchronization for each point (*M*02), and (3) design of a four-bar mechanism for tracking a trajectory delimited by a sequence of ten pairs of precision points (*M*03). The first swim favored exploration of the search space by using locations of other bacteria, while the second swim promoted fine movements in the vicinity of the bacterium position by using small step size values.

TS-MBFOA was analyzed in four experiments, where it was found that the two swims, unlike those of the other two BFOA-based algorithms, provided a larger number of better solutions along the search, even in its last cycles. Moreover, TS-MBFOA was able to consistently generate feasible solutions in the three optimization problems and its final results clearly outperformed those of MBFOA and IMBFOA because of its ability to avoid local optimum solutions. Furthermore, TS-MBFOA was able to obtain, with a single set of parameter values, competitive results in the three synthesis problems, with respect to one evolutionary algorithm designed for mechanical design optimization which required being fine-tuned for each synthesis problem. TS-MBFOA also found a similar competitive result for problem *M*01 but with less evaluations than the DE-based approach. Finally, from a mechanical point of view, the best solutions obtained by TS-MBFOA for the three optimization problems were highly competitive, suitable, and better than those found by MBFOA and IMBFOA.

The future work consists in revisiting the design of TS-MBFOA for studying the sensitivity to some of its parameters and trying to adapt their values. Finally, optimization problems of other mechanisms will be stated and solved.

## Figures and Tables

**Algorithm 1:**
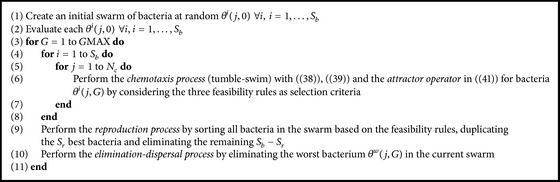
MBFOA. Input parameters are number of bacteria *S*_*b*_, chemotaxis loop limit *N*_*c*_, number of bacteria for reproduction *S*_*r*_ (usually *S*_*r*_ = *S*_*b*_/2), scaling factor *β*, percentage of initial stepsize *R*, and number of cycles (generations) *G*MAX.

**Algorithm 2:**
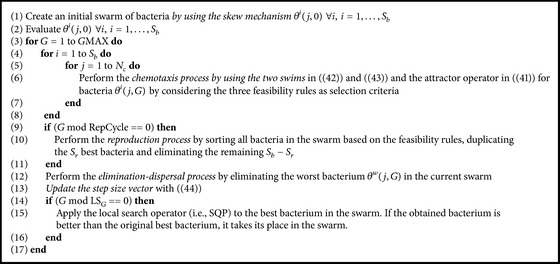
IMBFOA pseudocode. Input parameters are number of bacteria *S*_*b*_, chemotaxis loop limit *N*_*c*_, number of bacteria for reproduction *S*_*r*_, scaling factor *β*, the reproduction cycle RepCycle, the number of cycles *G*MAX, the local search frequency LS_*G*_, *τ* and *υ* for the search direction, and ss for the skew mechanism.

**Algorithm 3:**
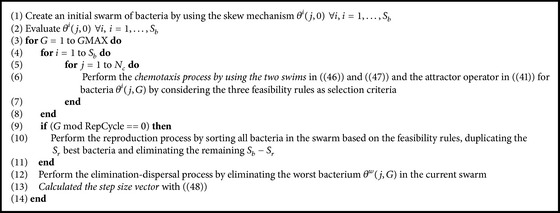
TS-MBFOA pseudocode. Input parameters are number of bacteria *S*_*b*_, chemotaxis loop limit *N*_*c*_, scaling stepsize *R*, number of bacteria for reproduction *S*_*r*_, scaling factor *β*, the reproduction cycle RepCycle, ss for the skew mechanism, and the number of cycles *G*MAX.

**Table 1:** Sign of radical in relation to the type of mechanism.

Configuration	*θ*_3_	*θ*_4_
Open	+	$-\sqrt{\;}$
Crossed	$-\sqrt{\;}$	+

**Table 2:** Pairs of precision points for case study 3.

Pair	*C*_1*d*_	*C*_2*d*_
1	(1.768, 2.3311)	(1.9592, 2.44973)
2	(1.947, 2.6271)	(2.168, 2.675)
3	(1.595, 2.7951)	(1.821, 2.804)
4	(1.019, 2.7241)	(1.244, 2.720)
5	(0.479, 2.4281)	(0.705, 2.437)
6	(0.126, 2.0521)	(0.346, 2.104)
7	(−0.001, 1.720)	(0.195, 1.833)
8	(0.103, 1.514)	(0.356, 1.680)
9	(0.442, 1.549)	(0.558, 1.742)
10	(1.055, 1.905)	(1.186, 2.088)

**Table 3:** Main features of each four-bar synthesis problem. Max_Evals is the maximum number of evaluations allowed per each of the problems, *n* is the number of design variables, and *c* is the number of constraints.

Problem	Max_Evals	*n*	*c*
*M*01	500,000	15	5
*M*02	100,000	6	4
*M*03	500,000	19	5

**Table 4:** Parameter values for the three BFOA-based compared algorithms. “—” indicates that the corresponding parameter is not required by the algorithm located in the column.

Parameter	MBFOA	IMBFOA	TS-MBFOA
*S*_*b*_	40	20	60
*N*_*c*_	24	20	10
*R*	1.2*E* − 02	—	5.00*E* − 03
*S*_*r*_	1	2	1
*β*	1.75	1.5	1.75
RepCycle	—	100	100
*G*MAX	Value to reach Max_Evals	Value to reach Max_FEs	Value to reach Max_FEs
LS_*G*_	—	1 and (*G*MAX/2) generations	—
*υ*	—	0.15	—
*τ*	—	−0.25	—
ss	—	8	8

**Table 5:** Feasible rate obtained by MBFOA, IMBFOA, and TS-MBFOA on 30 independent runs.

Prob.	MBFOA	IMBFOA	TS-MBFOA
*M*01	100%	100%	100%
*M*02	100%	100%	100%
*M*03	100%	100%	100%

**Table 6:** Statistical results obtained in 30 independent runs by MBFOA, IMBFOA, and TS-MBFOA when solving the three four-bar synthesis problems. Best results are remarked in boldface. All differences are significant based on the 95%-confidence Wilcoxon test.

Problem	Stat	MBFOA	IMBFOA	TS-MBFOA
*M*01	Evaluations	500,000	500,000	500,000
	Best	1.20*E* + 00	1.21*E* − 02	1.26**E** − 29
	Average	2.50*E* + 01	3.38*E* + 01	2.40**E** − 02
	Std.	2.57*E* + 01	6.93*E* + 01	9.15**E** − 02

*M*02	Evaluations	100,000	100,000	100,000
	Best	0.002997125	0.003726955	**0.002628079**
	Average	3.69*E* + 00	4.34*E* − 03	2.63**E** − 03
	Std.	2.98*E* − 04	9.29*E* − 04	1.31**E** − 17

*M*03	Evaluations	500,000	500,000	500,000
	Best	0.5630512	3.537282325	**0.2750193**
	Average	1.66*E* + 01	1.35*E* + 01	1.07**E** + 00
	Std.	2.72*E* + 01	1.03*E* + 04	1.10**E** + 00

**Table 7:** Best solution obtained by MBFOA, IMBFOA, and TS-MBFOA in the run located in the median value out of 30 independent runs. Best values are remarked in boldface.

Algorithm	*M*01	*M*02	*M*03
MBFOA	1.53*E* + 01	4.10*E* − 03	1.26*E* + 01
IMBFOA	1.51*E* + 02	3.93*E* − 03	7.75*E* + 01
TS-MBFOA	1.21**E** − 04	2.63**E** − 03	4.80**E** − 01

**Table 8:** Statistical results obtained in 30 independent runs by TS-MBFOA and the DE-based approach when solving the three four-bar synthesis problems. Best results are remarked in boldface. No significant differences were found based on the 95%-confidence Wilcoxon test.

Problem	Stat	TS-MBFOA	DE
*M*01	Evaluations	500,000	750,000
	Best	1.26217**E** − 29	1.26218**E** − 29
	Average	2.40*E* − 02	1.99**E** − 03
	Std.	9.15*E* − 02	5.39**E**0 − 03

*M*02	Evaluations	100,000	100,000
	Best	**0.002628079**	**0.002628079**
	Average	2.63**E** − 03	2.63*E* − 03
	Std.	1.31**E** − 17	4.473*E* − 10

*M*03	Evaluations	500,000	500,000
	Best	0.2750193	**0.274968745**
	Average	1.07**E** + 00	1.31*E* + 00
	Std.	1.10**E** + 00	3.27*E* + 00

**Table 9:** Best feasible solutions found by MBFOA, IMBFOA, TS-MBFOA, and the DE-based algorithms in each four-bar synthesis problem.

Variables	*M*01				*M*02				*M*03			
	MBFOA	IMBFOA	TS-MBFOA	DE	MBFOA	IMBFOA	TS-MBFOA	DE	MBFOA	IMBFOA	TS-MBFOA	DE
*x*_1_	56.96046969	49.93205601	24.0706441	37.35322401	19.59633374	27.35371004	14.31454786	14.31436975	38.12083651	18.60412365	2.632965423	2.614591501
*x*_2_	4.060834527	6.690293689	7.250066369	8.414037276	2.140600536	2.083629796	2.211165812	2.211165657	0.774854018	0.113660019	1.056973251	1.034801583
*x*_3_	8.923787074	19.2082537	20.94483824	27.79863521	18.56633857	22.85644937	14.31454786	14.3143696	19.29477637	5.578117599	1.763888952	1.826884381
*x*_4_	56.08270471	43.78525807	22.57639254	37.00803944	18.5842461	23.02591436	14.31454786	14.31436974	24.68930882	14.30112295	2.205482703	2.207891726
*x*_5_	− 25.83505865	− 11.38723361	31.53789197	37.61267067	2.239379687	2.286600675	2.174361597	2.174363923	8.333794141	− 11.78460943	1.206113171	1.250924301
*x*_6_	− 29.75007805	− 41.16507909	16.20250155	16.97308561	0.023572299	0.153197064	0.022209768	0.022212213	− 6.463530675	− 32.31550869	0.363242643	0.447340178
*x*_7_	4.267457973	4.28844377	4.156218625	3.966832786					5.93886	0.033123647	5.813750828	5.826803696
*x*_8_	54.8103088	55.71366791	− 7.286682385	− 9.678596639					− 8.625076191	− 8.396013249	0.084076204	0.099169603
*x*_9_	36.62395408	52.75753663	52.85173965	59.18270796					6.384329872	35.28021924	1.437990445	1.328798537
*x*_10_	1.120012858	0.082820007	1.24	1.717902384					0.038575927	0.029010814	0.396045164	0.410281221
*x*_11_	1.362491164	0.640982496	2.309698447	2.451658775					0.42340301	0.053824757	1.029004626	1.039364984
*x*_12_	1.621239336	0.964618395	2.879833244	2.966271497					0.971029032	0.588872867	1.625114804	1.650008311
*x*_13_	1.855988457	1.242039407	3.422015391	3.464656513					1.704933689	0.863809971	2.219097833	2.260034392
*x*_14_	2.11850087	1.505902613	3.997488978	4.013579749					2.378218281	0.882403592	2.803908752	2.865981127
*x*_15_	2.453816162	1.774143421	5.243554444	5.124608233					3.063472839	1.764509265	3.403882079	3.490250618
*x*_16_									3.67844246	2.024993472	4.09753211	4.163941276
*x*_17_									3.938125257	2.860256871	4.853550562	4.905546281
*x*_18_									4.286898695	4.250445558	5.380182303	5.416480223
*x*_19_									5.042731775	4.584225722	6.052924519	6.06760838
$f(\vec{x})$	1.206824913	0.012052859	1.26217**E** − 29	1.26218**E** − 29	0.002997125	3.73*E* − 03	**0.002628079**	**0.002628079**	0.5630512	3.537282325	0.275019399	**0.274968745**
